# The Education of Family Members and Older Adult Cognitive Health: Differences Across Racial Groups

**DOI:** 10.1093/geroni/igab046.2008

**Published:** 2021-12-17

**Authors:** Sindhu Vasireddy, Jenjira Yahirun, Mark Hayward

**Affiliations:** 1 International Institute for Population Sciences, India, Hyderabad, Telangana, India; 2 Bowling Green State University, Bowling Green State University, Ohio, United States; 3 University of Texas at Austin, University of Texas at Austin, Texas, United States

## Abstract

Education is a strong predictor of cognitive health among older adults, and recent research indicates that apart from one’s own education, the educational resources of family members also play a crucial role in shaping cognitive health over the life course. We add to this literature by investigating whether the advantages of highly educated family members matter for both Blacks and Whites in the U.S. Specifically, we ask whether the resources of family members-parents and offspring-partially explain the racial gap in both the prevalence and incidence of cognitive health across both groups. For this, we employ logistic regression models to examine the prevalence of cognitive impairment at baseline, and discrete-time event history models to assess the incidence of cognitive impairment, using data from the Health and Retirement Study (HRS) for the years ranging from 2000 to 2012. Preliminary results indicate that at the baseline, respondent’s own education is predictive of cognitive impairment among Whites, but not Blacks. Once respondent-level health conditions and behaviors are taken into consideration, parental or offspring education is not associated with the prevalence of cognitive impairment. For respondents who are not impaired at the baseline, our results from the incidence models align with the “adjacent generations” literature suggesting that both parental and offspring education has a salient positive effect on later-life cognitive health. However, we find notable differences across groups to suggest that the education of parents and offspring may play a larger role in mitigating cognitive decline among African Americans, compared to Whites.

